# Evaluation of cell disruption technologies on magnetosome chain length and aggregation behaviour from *Magnetospirillum gryphiswaldense* MSR-1

**DOI:** 10.3389/fbioe.2023.1172457

**Published:** 2023-05-04

**Authors:** Marta Masó-Martínez, Benjamin Fryer, Dimitri Aubert, Benjamin Peacock, Rebecca Lees, Graham A. Rance, Michael W. Fay, Paul D. Topham, Alfred Fernández-Castané

**Affiliations:** ^1^ Energy and Bioproducts Research Institute, Aston University, Birmingham, United Kingdom; ^2^ NanoFCM Co., Ltd., Nottingham, United Kingdom; ^3^ Nanoscale and Microscale Research Centre (nmRC), University of Nottingham, Nottingham, United Kingdom; ^4^ Aston Institute of Materials Research, Aston University, Birmingham, United Kingdom

**Keywords:** magnetic nanoparticles, magnetotactic bacteria, biomanufacturing, nano-flow cytometry, process analytical technology

## Abstract

Magnetosomes are biologically-derived magnetic nanoparticles (MNPs) naturally produced by magnetotactic bacteria (MTB). Due to their distinctive characteristics, such as narrow size distribution and high biocompatibility, magnetosomes represent an attractive alternative to existing commercially-available chemically-synthesized MNPs. However, to extract magnetosomes from the bacteria, a cell disruption step is required. In this study, a systematic comparison between three disruption techniques (enzymatic treatment, probe sonication and high-pressure homogenization) was carried out to study their effect on the chain length, integrity and aggregation state of magnetosomes isolated from *Magnetospirillum gryphiswaldense* MSR-1 cells. Experimental results revealed that all three methodologies show high cell disruption yields (>89%). Transmission electron microscopy (TEM), dynamic light scattering (DLS) and, for the first time, nano-flow cytometry (nFCM) were employed to characterize magnetosome preparations after purification. TEM and DLS showed that high-pressure homogenization resulted in optimal conservation of chain integrity, whereas enzymatic treatment caused higher chain cleavage. The data obtained suggest that nFCM is best suited to characterize single membrane-wrapped magnetosomes, which can be particularly useful for applications that require the use of individual magnetosomes. Magnetosomes were also successfully labelled (>90%) with the fluorescent CellMask™ Deep Red membrane stain and analysed by nFCM, demonstrating the promising capacity of this technique as a rapid analytical tool for magnetosome quality assurance. The results of this work contribute to the future development of a robust magnetosome production platform.

## 1 Introduction

Chemically-synthesized magnetic nanoparticles (MNPs) have been widely studied for their potential application in nanomedicine and biotechnology (Wu et al., 2019). However, synthetic MNPs face various drawbacks, such as high toxicity, lack of biocompatibility and harsh conditions required for their synthesis (Nanda et al., 2020). Some of these issues can be remediated by adding extra steps in the synthesis process, but these often come at the expense of production complexity and increasing costs. Therefore, research interests are shifting towards finding more efficient and sustainable biomanufacturing approaches to synthesize biocompatible MNPs.

The discovery of the biological MNPs, termed ‘*magnetosomes’* back in the 1970s, attracted the attention of the scientific community for their potential to replace synthetic MNPs (Blakemore, 1975). Magnetosomes are magnetic nanoparticles within the size range of 35–120 nm and are naturally produced by magnetotactic bacteria (MTB) (Faivre and Schüler, 2008). Usually, they are arranged in a needle-like chain and the main attributed function of the magnetosome chains is to act as a geomagnetic navigation systems. A typical magnetosome composition consists of an inorganic core (Fe_3_O_4_ or Fe_3_S_4_) enveloped in a lipid membrane bilayer containing transmembrane proteins (Grünberg et al., 2004). Some of the unique properties of magnetosomes include their narrow size distribution, high crystal purity, high heating capacity, facile functionalization, and high biocompatibility. These properties collectively make magnetosomes an attractive alternative to synthetic MNPs with great potential for biomedical, biotechnological and environmental application (Vargas et al., 2018). Unfortunately, the ability to implement a robust large-scale magnetosome biomanufacturing system has proven to be challenging despite the best efforts to enhance magnetosome yield (Yang et al., 2013; Fernández-Castané et al., 2018; Riese et al., 2020). The future widespread application of industrial magnetosome biomanufacturing is currently hindered by: (i) the limited understanding of magnetosome biomineralization; (ii) the identification of MTB growth and physiological bottlenecks (Moisescu et al., 2014; Fernández-Castané et al., 2017; Abdelrazig et al., 2020); (iii) the ability to produce magnetosome batches with the same structure, chain length and functionality (*i.e.*, batch-to-batch reproducibility); and (iv) the development of characterization techniques allowing rapid assessment of magnetosomes preparation.

Magnetosomes have the potential to become the next-generation of therapeutic agents but there is a critical need to scale-up the biomanufacturing process, whilst achieving homogeneous chain length preparation and preserving the envelope of embedded transmembrane proteins that can be used for functionalization purposes. The first step in the downstream process that compromises the size of magnetosome chains is cell disruption. Different lab-scale mechanical cell disruption techniques such as ultrasonication (Sun et al., 2008; Raguraman et al., 2020), French Press (Grünberg et al., 2004; Xiang et al., 2007) or high-pressure homogenization (Guo et al., 2011; Tang et al., 2012) are commonly used for MTB disruption thus rendering magnetosomes accessible for recovery. However, very few studies have been conducted to systematically compare different cell disruption treatments, and thus evaluate their efficiency and impact on the length and integrity of the isolated magnetosome chains (Nakamura et al., 1991; Alphandéry et al., 2013). In this study, we employ three nanoparticle (NP) characterization techniques, namely, transmission electron microscopy (TEM), dynamic light scattering (DLS) and nano-flow cytometry (nFCM) to evaluate the effect of three different cell disruption techniques on magnetosome chains. The combination of such complementary techniques allows one to obtain a holistic picture of the composition of the magnetosome preparation as each technique has its own advantages and disadvantages. For instance, TEM offers high size resolution but low throughput, whereas the opposite is observed in DLS analysis. nFCM was used for the first time to characterize magnetosomes and we have evaluated its potential to become a rapid quality assurance technique for magnetosome preparation. The combination of light scattering and fluorescence detection from nFCM allows the facile collection of data to inform the particle size distribution, particle concentration and identification of sub-populations labelled with fluorescent markers, which makes nFCM a very powerful and innovative technique (Lian et al., 2019).

## 2 Materials and methods

### 2.1 Bacterial strains and culture conditions

*Magnetospirillum gryphiswaldense* (MSR-1) was obtained from Deutsche Sammlung von Mikroorganismen und Zellkulturen GmbH (DSMZ, Germany). MSR-1 was grown in a 5-L Biostat B (Sartorius Stedim UK Ltd., Surrey, UK) bioreactor following a pH-stat strategy described elsewhere (Fernández-Castané et al., 2018). Briefly, MSR-1 cells were grown at 30°C, under microaerobic conditions (pO_2_ < 1%.), in flask standard medium (FSM) without iron citrate at a pH of 7. The pH was maintained by automated addition of an acidic feeding solution. Once the culture reached stationary phase, MSR-1 cells were harvested by centrifugation (4,000 rpm, 20 min, 4°C) in a Heraeus Multifuge X1R centrifuge (Thermo Scientific, Massachusetts, United States). The supernatant was removed and the pellet was stored at −80°C until further use.

### 2.2 Cell disruption

MSR-1 biomass was thawed overnight at 4°C and cells were disrupted using three different methods: (i) enzymatic disruption (EZ); (ii) sonic probe disruption (SP); and (iii) high-pressure homogenization (HPH). Prior to disruption, MSR-1 biomass for HPH and SP samples were suspended in a HEPES-EDTA buffer solution (50 mM HEPES, 5 mM EDTA, pH 7.4) to a final concentration of 10% (w/v). High-pressure homogenizer disruption was conducted using a bench top CF1 Cell Disruptor (Constant Systems Ltd., Daventry, Northants, UK). Suspended cells were disrupted in a single pass at 10 kpsi at 4°C (Li, 2018). Sonic probe disruption was completed by sonicating the cells for 20 min at 4°C using 70% amplitude with a pulse setting of 1 s on/1 s off using the Fisherbrand™ Model 120 Sonic Dismembrator (500 W, 20 kHz) (Supplementary Figure S1). The enzymatic disruption protocol was adapted from the manufacturer’s instructions. Briefly, cells were suspended (10% (w/v)) in B-PER™ Complete Bacterial Protein Extraction Reagent (Thermo Scientific), which contained a mild non-ionic detergent and lysozyme, for 60 min at 25°C at 100 rpm.

### 2.3 Magnetosome purification

Regardless of the methodology employed to disrupt MSR-1 cells, magnetosomes were recovered following the same purification protocol adapted from Fernandez-Castane et al. (Fernández-Castané et al., 2021). The disrupted cell suspensions were subjected to magnetic separation using a NdFeB N45 magnet by performing at least a series of 10 washes. The magnet was placed vertically against the tube containing the cell homogenate for 30–45 min at 4°C, except for the first wash which was left overnight. The magnet attracts any magnetic material and cell debris remains in the suspension or sedimented at the bottom of the tube. Using a pipette, the MSR-1 cell debris fraction was carefully removed before adding 20 mL of fresh HEPES-EDTA buffer. Then, the solution was resuspended and left with the magnet for another 30–45 min. The same procedure was repeated 10 times except only 6 mL of HEPES-EDTA buffer was used to resuspend the magnetosomes in the final wash. To remove remaining impurities, ultracentrifugation of the recovered magnetosomes was performed using a Ti 70.1 Rotor on the Optima™ L-80 XP Ultracentrifuge (Beckman Coulter). 1 mL portions of the recovered magnetosomes were layered onto a 5 mL 60% (w/v) sucrose cushion using 10.4 mL Polycarbonate Bottles with Cap Assembly (Model 355603, Beckman Coulter, Indianapolis, United States) and ultracentrifuged at 45,000 rpm, 4°C for 2.5 h. After centrifugation, the ‘light’ sucrose top phase was carefully removed using a Pasteur pipette, and the ‘heavy’ fraction at the bottom containing magnetosomes was resuspended in PBS (pH 7.4). Samples were subsequently collected and stored at 4°C in a reducing environment to avoid magnetosome oxidation until further analysis.

### 2.4 Treatment of purified magnetosomes

Purified magnetosomes were analysed by TEM, DLS and nFCM. A fraction of these purified magnetosome solutions were split and used to obtain (i) single magnetosomes with intact membrane and (ii) membrane-stripped magnetosomes. To obtain single magnetosomes, the preparations were vortexed for 1 min and sonicated for 5 min in a Clifton SW1H Ultrasonic bath (200 W, 37 kHz). Membrane-stripped magnetosomes were generated as described elsewhere (Hamdous et al., 2017). Briefly, purified magnetosomes were treated with a series of detergents and organic solvents and after each treatment, magnetosomes were isolated from non-magnetic organic debris using a NdFeB N45 magnet as follows: (i) 20 s sonication of PBS-resuspended magnetosomes in an ultrasonic bath; (ii) overnight resuspension of magnetosomes in a 1% SDS solution at 60°C; (iii) sonication of magnetosomes in phenol (pH 8) solution for 2 h at 60°C in an ultrasonic bath; (iv) chloroform-resuspension of magnetosomes for 2 h at 60°C; and (v) sonication of magnetosomes resuspended in a 1 M NaOH solution for 1 h at 60°C in an ultrasonic bath. Subsequently, membrane-stripped magnetosomes were resuspended in PBS.

### 2.5 Transmission electron microscopy

Transmission electron microscopy (TEM) was used to image magnetosome chains. Magnetosome preparations (2 µL) were drop cast on a graphene oxide on lacey carbon 300 mesh copper supported grid (GOLC300Cu50, EM Resolutions, Sheffield, UK) and vacuum-dried before analysis. TEM images were obtained using a JEM-2100 F microscope (JEOL, Herts, UK) operating at 200 kV and equipped with a Gatan Orius CCD camera (Pleasanton, United States). To determine the length distribution of the purified magnetosome chains, 100 magnetosome chains per sample were selected randomly and the number of magnetosome units of each chain was counted. Magnetosome chains were also classified into three different categories: short (1-5 magnetosome units), medium (6–10 magnetosome units) and long chains (>11 magnetosome units). Other relevant parameters, such as mean values and cumulative frequency distributions were also calculated and analysed for each sample. From cumulative frequency data, relevant percentiles, P_0.5_ (L_50,_ median) and P_0.1_ (L_10_), were calculated. Microscope images of the pre-disrupted MSR-1 cells were analysed as described above.

### 2.6 Dynamic light scattering

Dynamic light scattering (DLS) was used for the characterization of the size distribution of the purified magnetosome solutions. The size of particles, as hydrodynamic diameter, is calculated from the translational diffusion coefficient related to the velocity of Brownian motion by using the Stokes-Einstein equation (Falke and Betzel, 2019). Each sample was analysed using a Zetasizer Nano-ZS (Malvern Panalytical, Worcs, UK) in a standard capillary cell with a backscattering angle of 173°. PBS was used as the suspension buffer (viscosity 0.8872 cP, 1.330 refractive index, 25°C). A total of 6 measurements per sample were performed and average size values are presented.

### 2.7 Determination of iron concentration

Inductively coupled plasma optical emission spectroscopy (ICP-OES, Thermo Scientific iCAP 7,000) coupled to a Teledyne CETAC ASX-520 Random Access Autosampler was used to determine the iron concentration of magnetosome preparations at a wavelength of 259.94 nm. 100 μL of purified magnetosome preparation were used and digested with 500 μL of nitric acid (70% v/v) solution. Samples were prepared in triplicate, incubated at 98°C for 2 h with shaking at 300 rpm prior to be analysed by ICP-OES.

### 2.8 Flow cytometry

Samples were taken before and after cell disruption, diluted in phosphate-buffered saline (PBS) solution and immediately analysed in a BD Accuri C6 Flow Cytometer (Becton, Dickinson and Company, Oxford, UK). Samples were stained with Syto^®^62 (0.4 μM), a permeant DNA dye, to stain MSR-1 cells to determine cell disruption efficiency. Syto^®^62 was excited with a 488 nm solid-state laser and detected through a 675/25 BP filter (FL4-A).

### 2.9 Nano-flow cytometry

A NanoAnalyzer U30 instrument (NanoFCM Inc., Nottingham, UK) equipped with dual 488/640 nm lasers and single-photon counting avalanche photodiode detection (SPCM APD) was used for simultaneous detection of side scatter (SSC) and fluorescence of individual particles. Bandpass filters allowed for collection of light in specific channels (SSC - 488/10; FL1–525/40; FL2–670/30). HPLC-grade water served as the sheath-fluid via a gravity feed, reducing the sample core stream diameter to ∼1.4 µm. Measurements were taken over a 1-min interval at a sampling pressure of 1.0 kPa, maintained by an air-based pressure module. All samples were diluted in PBS to attain a particle count within the optimal range of 2,000–12,000/min. During sample acquisition, the sample stream is fully illuminated within the central region of the focused laser beam, resulting in approximately 100% detection efficiency, which leads to accurate particle concentration measurement via single-particle enumeration. The concentration of samples was determined by comparison to 250 nm silica nanoparticles of known concentration to calibrate the sample flow rate. Particle sizing was carried out according to standard operating procedures using a proprietary 4-modal silica nanosphere cocktail (NanoFCM Inc., S16M-Exo–68, 91, 113 and 155 nm). Using the NanoFCM software (NanoFCM Profession V1.8), a standard curve was generated based on the side scattering intensity of the different silica particle populations. In order to detect the whole particle size distribution of the different samples, the laser was set to 10 or 20 mW and the SS (side scatter) decay to either 10% (40–200 nm particle range) or 0.2% (200–1,000 nm particle range). Data processing was handled within the nFCM Professional Suite v1.8 software, with dot plots, histograms, and statistical data. Gating within the software allows for proportional analysis of subpopulations separated by fluorescent intensities with size distribution and concentration available for each sub-population. To homogenize magnetosome preparations, to avoid clumps and aggregates, and to obtain a uniform particle concentration, the solutions were subjected to 5 s vortexing and 10 s gentle sonication using a Clifton SW1H Ultrasonic bath.

#### 2.9.1 Magnetosome labelling

Purified and treated preparations of single magnetosomes were used to conduct this experiment. To avoid aggregates, samples were homogenized by vortexing and sonication as described above every time sample manipulation was required (e.g., diluting, staining). Purified magnetosome preparations were labelled using the fluorophore CellMask™ Deep Red (Invitrogen™, UK), which is a lipid membrane stain, adapting a protocol described elsewhere (Shentu et al., 2017; Bortot et al., 2022). To determine the optimal concentration of CellMask™ Deep Red, different fluorophore concentrations were used (1.25, 6.25, 20, 25, and 100 μg/mL). Incubation conditions were maintained constant: after dye was added, samples were incubated for 30 min at room temperature. After 15 min of incubation, samples were again briefly sonicated and vortexed. Three replicates were prepared per each staining condition.

### 2.10 Statistical analysis

Unless otherwise indicated, results are represented as means ± standard error of the mean. One-way analyses of variance (ANOVA) followed by Bonferroni’s post-test were performed to compare the different groups. The cut-off value for statistical significance was set at *p* < 0.05.

## 3 Results and discussion

In this study, the effect of cell disruption treatment on the length and integrity of the magnetosome chain was studied. Three different cell disruption treatments (EZ, SP and HPH) and three different magnetosome characterisation techniques (TEM, DLS and nFCM) were employed to determine magnetosome chain length from MSR-1 cells cultivated at relatively high cell densities.

### 3.1 Disruption efficiency

**FIGURE 1:**
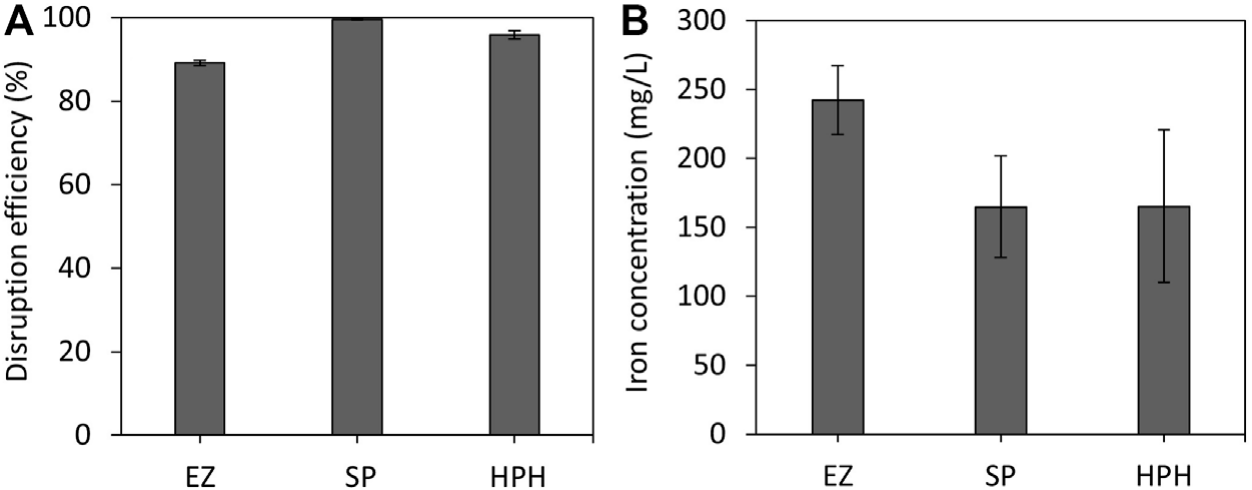
**(A)** *M. gryphiswaldense* MSR-1 cell disruption efficiencies of EZ, SP and HPH cell disruption treatments. **(B)** Estimation of iron concentration of purified magnetosome solutions. Note: EZ (enzymatic), SP (sonication probe) and HPH (high-pressure homogenizer) treatments.

Breaking down the cell membrane is necessary to release the intracellular contents and thus extract and isolate magnetosome chains. To determine the efficiency of each disruption treatment, cells were stained with Syto^®^62 and analysed by FCM. Efficient magnetosome recovery requires a high disruption efficiency, but to accomplish this, the magnetosome chain integrity might be compromised. Figure 1A shows that all three disruption techniques exhibit high efficiency (>89%) in terms of cell lysis. This is due to the fact that all three techniques and operation conditions were carried out under optimal conditions for efficient MSR-1 disruption. As no significant differences in cell disruption efficiencies were observed, other factors, such as time, scalability or availability of resources, must be considered and are further discussed in this work. EZ treatment displayed the lowest efficiency (89%) among all treatments. As expected, SP treatment resulted in the highest disruption efficiency (>99%) followed by HPH treatment (96%). Previous work done by Li and co-workers reported a similar disruption efficiency of 92% using a high-pressure homogenizer and also reported that it was easier to disrupt MSR-1 cells when they were harvested at the exponential phase rather than when cells were in stationary phase (Li, 2018). Cell wall strength, shape and size can play an important role in cell disruption efficiency, especially in high-pressure homogenisation (Kleinig and Middelberg, 1998).

Usually, ICP-OES or other spectroscopic techniques are commonly used to obtain a quantitative estimation of magnetosome content. In this case, the analysis of iron content of the purified magnetosome solutions using ICP-OES also provided information regarding the cell disruption efficiency. Figure 1B shows that magnetosome sample isolated using SP and HPH contain similar iron content, however, for those samples using EZ higher iron levels were observed. All steps after MSR-1 cell disruption were fixed to evaluate solely the effect of the disruption step. EZ disruption was the least efficient cell disruption method, which indicated that a higher number of cells remained intact after the disruption and purification processes compared with the other treatments. In fact, whole cells were observed in some of the EZ TEM images (Supplementary Figure S2) whereas the presence of whole cells was not observed in TEM images from SP and HPH samples. The presence of whole cells in the EZ magnetosome preparation could explain the superior iron levels in those samples. Until recently, it was believed that 99.5% of the intracellular iron content of MTB cells corresponded to magnetite crystals (Grünberg et al., 2004). However, recent studies estimated that only 25%–45% of the bulk cellular iron corresponded to magnetite (Amor et al., 2020; Berny et al., 2020). The remaining intracellular iron is believed to be used to carry out general biochemical reactions that require iron. Therefore, all the intracellular iron content of undisrupted cells in the EZ treatment that do not correspond to magnetite was added up to the final iron concentration.

The EZ commercial kit used contained lysozyme in combination with a mild non-ionic detergent. Lysozyme had previously been used to study periplasmatic and cytoplasmatic fractions in MTB cells (Noguchi et al., 1999; Suzuki et al., 2007), but very few studies have used this enzyme for magnetosome extraction (Nakamura et al., 1991). French press (Grünberg et al., 2004; Curcio et al., 2020), high-pressure homogenization (Tang et al., 2012; Amor et al., 2014; Fernández-Castané et al., 2021) or sonication (Kobayashi et al., 2006; Alphandéry and Chebbi, 2012) are more commonly used for disruption of MTB due to their simplicity and cost-effectiveness. The effect of these disruption techniques on magnetosome chains has not been studied in detail yet, but large aggregates have been observed when the magnetosome membrane is removed. Extensive ultrasonication time or enzymatic treatments can cause the loss of the magnetosome membrane (Nakamura et al., 1991; Alphandéry et al., 2011), which prevents a homogeneous particle distribution. Increasing the number of passes in HPH treatment also enhances chain breakage (Li, 2018). Very little has been reported in terms of systematic and comparative cell disruption studies in MTB, hence the importance of the work herein.

### 3.2 Effect of cell disruption method on magnetosome chain length and aggregation state

**FIGURE 2:**
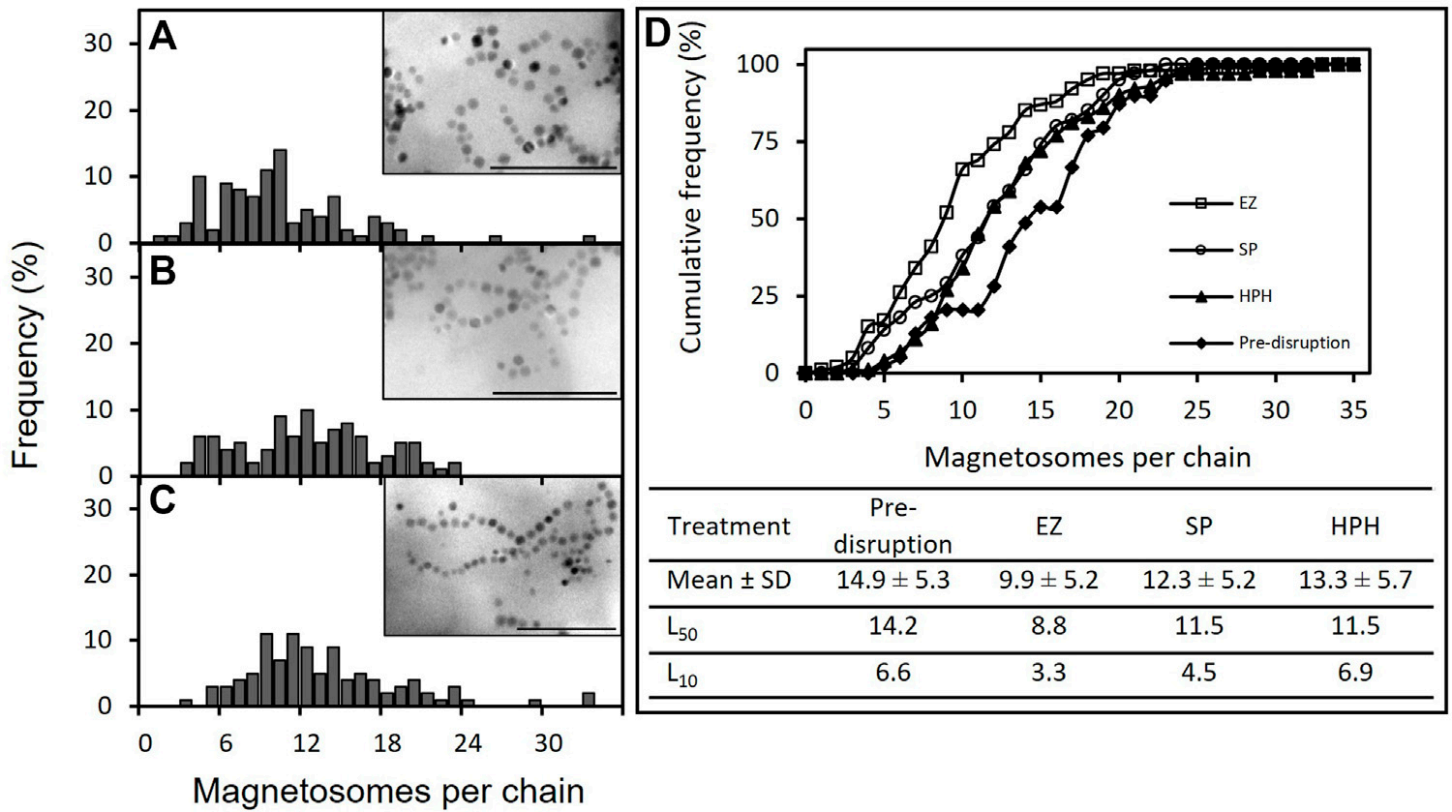
TEM analysis of purified magnetosome chains *of M. gryphiswaldense* MSR-1. **(A,–C)** Magnetosome chain length frequency distribution plots and TEM images of purified magnetosome preparations in which enzymatic treatment **(A)**, sonication **(B)** and high-pressure homogenizer **(C)** were employed to disrupt MSR-1 cells; **(D)** Cumulative frequency curves of magnetosome chain length before and after magnetosome purification. The table includes the mean values of the length of the magnetosome chains pre- and post-cell disruption, as well as relevant statistical parameters, L_50_ and L_10_. Note: EZ (enzymatic), SP (sonication probe) and HPH (high-pressure homogenizer) treatments. Scale bar: 500 nm.

The effect of cell disruption treatment on magnetosome chain length was first examined by TEM (Figure 2). Micrographs of the purified magnetosome solutions were analyzed by eye. Up to 100 magnetosome chains per treatment were selected and the number of magnetosome units in each chain was determined. The variation of the mean values of magnetosome chain length revealed the existence of significant differences among some of the disruption treatments (Figure 2D). EZ treatment generated statistically significant shorter magnetosome chains compared with the rest of the treatments (one-way ANOVA with Bonferroni test, *p* < 0.05). However, there are no significant differences between SP and HPH treatments or between HPH and pre-disrupted chains. As Figures 2A–C shows, the distributions of magnetosome chain sizes in all the disruption treatments are broad, displaying heterogeneity in the magnetosome preparations and the existence of different chain-size populations.

Table 1 classifies magnetosome chains in three different groups based on their length: short, medium, and long. Considering that 80% of the magnetosome chains were long before cell disruption, it can be observed that cell disruption caused chain cleavage in all three treatments as the prevalence of long chains was reduced by at least 15% in each case (Table 1). As a result of chain breakage, short chains which were not present in pre-disrupted MSR-1 cells were detected after cell disruption. HPH treatment seemed to be the technique that generated less damage to the chains, yielding the lowest short-chain frequency (4%) and the highest long-chain frequency (66%) (Alphandéry et al., 2013; Li, 2018; Fernández-Castané et al., 2021). The results also revealed that EZ treatment most significantly affected the chain integrity as only 34% of the chains were considered long and 17% short. These results suggest that the enzymatic cocktail used could be compromising the cell membrane as well as the magnetosome membrane. In addition, MamK, the actin-like protein that arranges magnetosomes in a chain, could also be affected by the EZ treatment as it has been seen that actin polymerization can be affected by the presence of detergents (Ujfalusi-Pozsonyi et al., 2010). The EZ kit used contained a mild non-ionic detergent that may contribute to MamK degradation, causing more magnetosome chain cleavage.

**TABLE 1:** Classification of magnetosome chains length frequency (%) into short, medium or long chains before (pre-disruption) and after magnetosome purification based on TEM analysis.

		Short	Medium	Long
Pre-disruption		0	20	80
Disruption treatment	EZ	17	49	34
	SP	14	24	62
	HPH	4	30	66

Cumulative frequency is used to determine the number of observations that lie above or below a particular value in a data set. Determination of relevant cumulative frequency percentiles such as L_50_ (median) and L_10_ are particularly useful in cases like this study in which the distribution of events is asymmetric, as these percentiles are less sensitive to extreme values and provides a more precise estimate of the size distribution. Cumulative frequency curves (Figure 2D) indicated that SP and HPH followed similar chain length distributions, whereas the EZ treatment deviated from them by displaying shorter magnetosome chains. SP and HPH L_50_ values showed no differences between them (11.5 magnetosome per chain), but smaller percentile values such as L_10_ revealed disparities in chain length between these two magnetosome preparations (4.5 and 6.9 magnetosomes per chain, respectively), which suggested the presence of higher quantities of short chains in the SP preparation. It is known that uncoated or partially coated magnetosomes are prone to self-aggregation (Kobayashi et al., 2006). Previous studies revealed extensive chain breakage and magnetosome agglomeration as a direct consequence of magnetosome ultrasonication over an extended period of time (Alphandéry and Chebbi, 2012; Fernández-Castané et al., 2021). Nakamura and co-workers compared ultrasonication and lysozyme disruption treatments by studying the particle sizes as well as magnetosome membrane thickness (Nakamura et al., 1991). TEM images revealed that the degree of chain aggregation in the EZ preparation was higher than in the SP preparation. In addition, magnetosome membrane thicknesses in lysozyme-treated magnetosomes were thinner than ultrasonicated magnetosomes, indicating the effect of the EZ treatment not only on MTB cells but also on the magnetosome membrane (Nakamura et al., 1991). Nakamura combined the lysozyme treatment with either the addition of SDS, which can denature some Mam proteins (Reynoldst and Tanfordt, 1970), or incubation with NaOH for 12 h, which can be time consuming. The MTB species used is not specifically identified in their study either, so to our knowledge, our study is the first one to use a lysozyme treatment on MSR-1 for cell disruption purposes.

**FIGURE 3:**
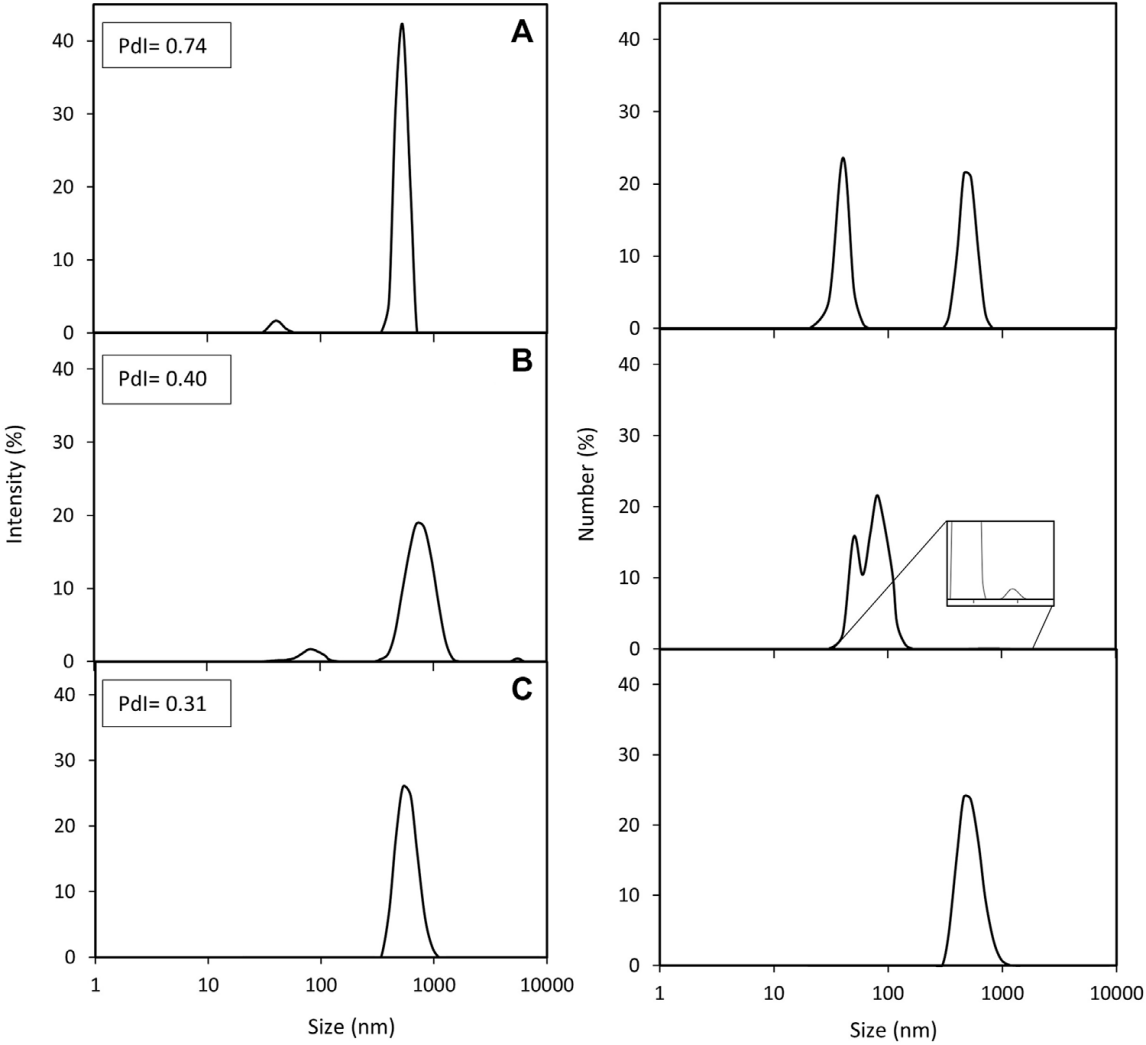
DLS analysis of purified magnetosome chains extracted from *M. gryphiswaldense* MSR-1. Intensity and number distributions of purified magnetosome solutions obtained from disrupting MSR-1 cells using either **(A)** enzymatic, **(B)** sonication or **(C)** high-pressure homogenization treatments. Polydispersity index (PdI) values for each magnetosome preparation are included.

Powerful techniques, such as TEM, allow the determination of the exact number of magnetosome crystals per chain and the study of the chain and crystal morphology. However, this technique only scrutinizes a tiny portion of the sample, hence there is a clear need to combine this with different techniques to obtain a broader picture. In this study we used DLS and nFCM to address the limitations of TEM analysis. One advantage of employing DLS is that this technique can analyse the whole preparation and study the particle behaviour in solution in a short period of time. DLS and TEM results are often complementary to each other but, given the two techniques fundamentally measure attributes such as size in different ways, they do not always directly correlate with one another. For instance, DLS provides an estimate of the average length/size of the chains as a hydrodynamic diameter; as this assumes a spherical morphology, it cannot readily account for the absolute shape of the chains (e.g., curved, straight, closed loops, agglomerates). Moreover, as they are in suspension, magnetosomes are free to move, rotate and interact with each other, providing an opportunity to study aggregation dynamics in real time (Szatanek et al., 2017). Figure 3 shows the intensity and number distributions of the three different magnetosome solutions. In both distributions, the same peaks can be observed. Number distributions emphasize the species with highest number of particles (usually smaller particles) whereas intensity distributions highlight the species with the largest scattering intensity (usually the larger particles), hence the difference in height of the peaks. Since the particle size distribution of the magnetosome preparations produced in this work is not narrow, the presence of larger particles, such as aggregates or long chains, will contribute to an increase in light scattering, shifting the measured particles size towards larger values (Souza et al., 2016). While analysis of HPH (Figure 3C) only resulted in one peak, EZ (Figure 3A) and SP (Figure 3B) bimodal size distributions indicate the presence of two magnetosome populations: one consisting of short chains or single magnetosomes and the other of long magnetosome chains (Gojzewski et al., 2012; Mannucci et al., 2014). The higher prevalence of short chains in EZ and SP treatments compared to HPH is consistent with TEM results (4% HPH, 14% SP, 17% EZ frequency of short chains) (Table 1). The polydispersity index (PdI) of a solution measures the uniformity of particle sizes in a solution. The high PdI of the EZ magnetosome preparation (PdI = 0.74) (Figure 3) reflects the existence of a broader size distribution and indicates that the peak corresponding to single magnetosomes might represent a much larger population of short magnetosome chains than the one from SP treatment. Our results show that the chain integrity and aggregation can be tuned as a function of the cell disruption technique, providing a range of MNPs tailored for a specific application. For some applications in which single magnetosomes are preferred or when the magnetosome membrane needs to be stripped to cover the magnetite crystals with different polymers, preserving chain length is less relevant (Yoshino et al., 2008; Alphandéry et al., 2017; Hamdous et al., 2017). Therefore, the chain cleavage effect of EZ treatment is not concerning in these cases. For other applications, such as alternative magnetic field cancer therapy (Alphandéry et al., 2011) or magnetic hyperthermia treatment of tumours (Alphandéry et al., 2013), magnetosome chains are more effective than using single magnetosomes. Other studies reported the benefits of using clustered magnetosomes as contrast agents for magnetic particle imaging instead of linear chains (Makela et al., 2022).

Scalability, time consumption and the cost of the different cell disruption treatments, among other factors, are also important elements to consider when determining the optimal magnetosome downstream processing for each intended application. SP is typically used in laboratories because of its simplicity, low operational costs and ease of use to treat small volumes (Gomes et al., 2020). HPH treatments are more effective and easier to adapt for large-scale processes but usually require higher energy input (Bernaerts et al., 2019), contrary to EZ treatments, which require less energy consumption. The possibility of scaling-up is feasible for all three technologies in terms of industrial equipment; however, SP is usually less scalable and EZ generates higher costs for long term use on an industrial scale (Üstün-Aytekin et al., 2016). One advantage of the EZ treatment over SP and HPH is that heat is not generated while operating and does not require a refrigeration system to avoid sample damage (Tangtua, 2014). At smaller scale, time is not that detrimental as working with smaller volumes and lower cell concentrations usually ease the process. However, time costs and the amount of personnel needed to operate the system is more important at industrial scale when choosing the preferred disruption method as it will significantly contribute to the final costs of the process.

### 3.3 Suitability of nano-flow cytometry analysis for the characterisation of magnetosome preparations

Conventional characterization techniques such as TEM and DLS are powerful and commonly used for nanoparticle characterization studies, yet they can be time consuming and have a low detection sensitivity, respectively. In this study, we assessed nano-flow cytometry as a tool for rapid characterisation of magnetosome preparations with high sensitivity, which, to the best of our knowledge, has never been used before. The detection of nanoparticles by scatter and fluorescence allows the capture of real time data of nanoparticle size populations, size distribution, concentration and biochemical properties.

#### 3.3.1 Effect of ultrasonication on magnetosomes concentration

Magnetosome preparations were diluted to be within the optimal particle count range of 2000–12,000/min. Due to the nature of the samples, dilution to suitable and uniform concentrations proved to be difficult. If the solution is not properly resuspended when diluting, magnetosome aggregates can be taken from the sample, and later when they are disaggregated the concentration increases. To solve this problem, a protocol was established in which samples were vortexed for 5 s, sonicated for 10 s, and immediately diluted or analysed before aggregates started to form. Following this methodology, data obtained for particle concentration was more stable and reproducible. If samples are sonicated too many times or for too long, it can affect the integrity of the magnetosome membrane, which can lead to the formation of more aggregates and cause constant changes in sample concentration (Kobayashi et al., 2006).

#### 3.3.2 nFCM analysis of the effect of cell disruption on magnetosome chains

**FIGURE 4:**
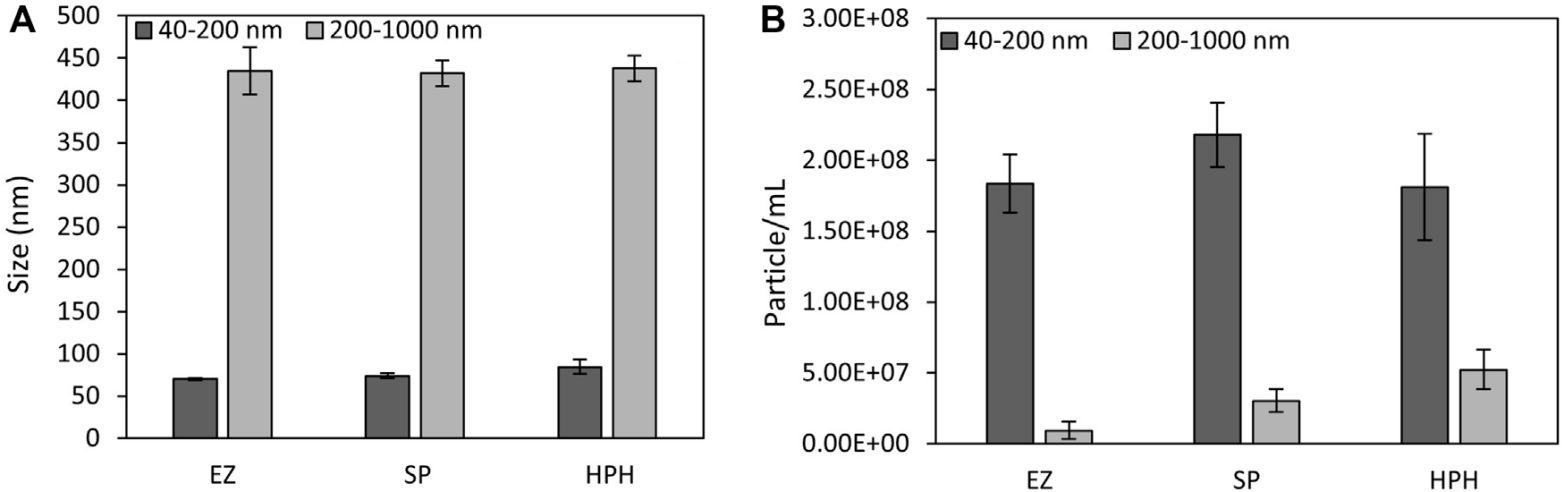
**(A)** *M. gryphiswaldense* MSR-1 magnetosomes chain size mean values and **(B)** particle concentrations of magnetosome preparations measured by nFCM using a smaller (40–200 nm) and larger (200–1,000 nm) particle detection range. EZ (enzymatic), SP (sonication probe) and HPH (high-pressure homogenizer) treatments.

The effect of cell disruption treatment on magnetosome chain length and the particle concentration of each preparation was examined by nFCM. To detect all magnetosome chain sizes, different laser settings were required to distinguish smaller particles (40–200 nm) from larger particles (200–1,000 nm). nFCM particle concentration results revealed a vast difference in sample concentration when the preparations were analysed using the small or the large particles detection ranges (Figure 4B). For the smaller particles range, the concentrations obtained were much higher than for the larger particles. EZ, SP and HPH samples were 20, 7 and 3 times more concentrated when measured using the smaller particles detection range than when using the large particles range, respectively. The three-fold particle concentration difference of the HPH preparation compared to the 20-fold difference of the EZ sample indicated the presence of a higher amount of short magnetosome chains from the EZ conditions, which matched the 4% and 17% short magnetosome chains measure from TEM results, respectively (Table 1). However, the EZ preparation showed the lowest concentrations among all three treatments. Magnetosomes with damaged or stripped membranes tend to aggregate to form clusters, which are not suitable for nFCM analysis (Kobayashi et al., 2006; Alphandéry and Chebbi, 2012). When analysing membrane-stripped magnetosomes, clusters larger than 1 µm were observed and impeded the analysis with nFCM. Therefore, if EZ cell disruption affects not only the cell membrane but the magnetosome membrane too, membrane-stripped magnetosomes cannot be detected during nFCM analysis, which explains the decrease in the overall EZ particle concentration (Nakamura et al., 1991).

**FIGURE 5:**
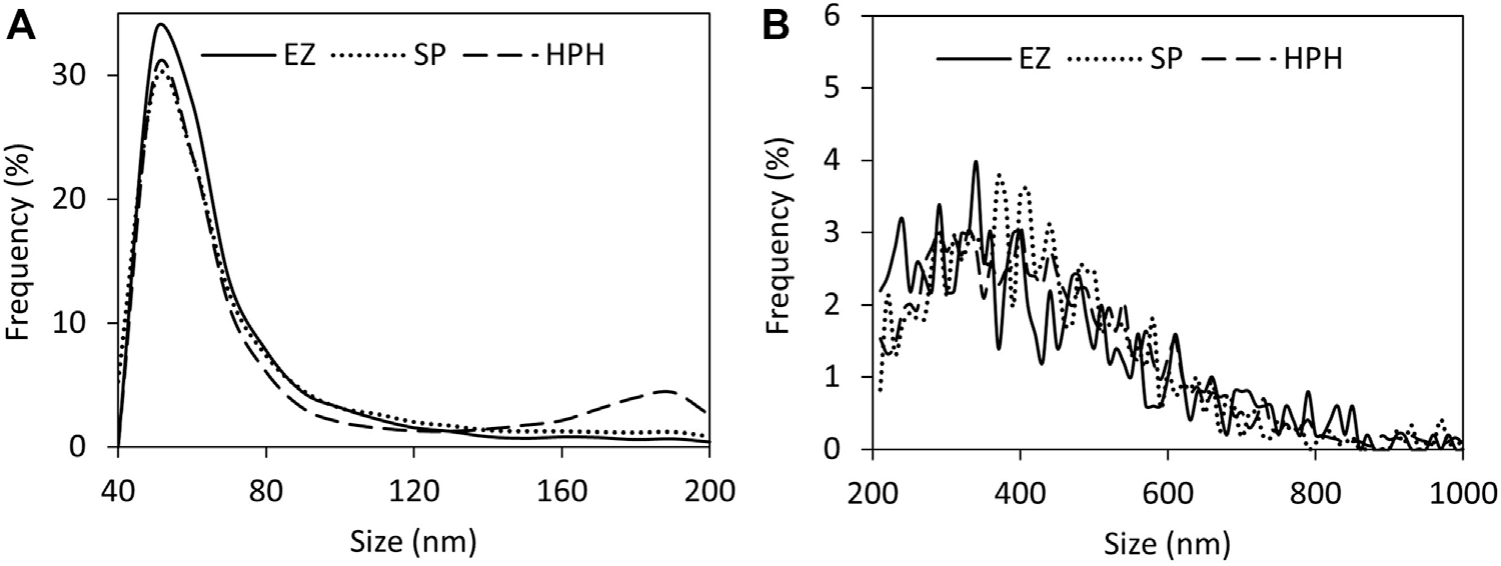
nFCM analysis of MSR-1 magnetosome chain size distributions using different detection ranges for **(A)** smaller particles (40–200 nm) and **(B)** larger particles (200–1,000 nm). Note: enzymatic (EZ), sonication (SP) or high-pressure homogenization (HPH) treatments.

Particle size distribution plots (Figure 5) did not show significant differences among the three different disruption treatments. Only the small particles plots (Figure 5A) exhibited a clear peak around 60 nm, which likely corresponded to single magnetosomes. Mean size values (Figure 4A) did not show significant differences among the treatments either (one-way ANOVA, *p* < 0.05). The results suggest the better suitability of nFCM to characterize single magnetosomes than longer magnetosome chains. In addition, the necessity of having to acquire two size ranges (40–200 nm and 200–1,000 nm) and the sample preparation protocol may biase the actual size distributions as well as the particle concentrations of the different magnetosome preparations, which may represent a disadvantage over other similar techniques such as Nanoparticle Tracking Analysis (NTA) (Fernández-Castané et al., 2021). nFCM is a reliable technique to determine nanoparticle concentrations but due to the heterogeneity in the magnetosome chain length and the nature of the technology, this technique is not suited to characterize the size distribution of untreated magnetosome preparations. Individual membrane-wrapped magnetosomes have a larger surface area-to-volume ratio, which might be an advantage for certain applications (Vaghari et al., 2015). Table 2 shows a summary of all the results obtained using the different characterization and disruption techniques.

**TABLE 2:** Summary of the magnetosome preparation characterization results for each disruption technique.

		EZ		SP		HPH	
Disruption efficiency (%)		89.2 ± 0.6		99.6 ± 0.2		95.9 ± 1	
TEM	MS per chain	9.9 ± 5.2		12.3 ± 5.2		13.3 ± 5.7	
DLS	PdI	0.74		0.40		0.31	
	Intensity Peak (nm)	Peak 1	Peak 2	Peak 1	Peak 2	Peak 1	
		40	530	80	830	530	
nFCM	Particle Mean Size (nm)	40–200 nm	200–1000 nm	40–200 nm	200–1000 nm	40–200 nm	200–1000 nm
		74.34 ± 1.36	406.37 ± 36.75	77.11 ± 0.66	404.63 ± 15.97	100.44 ± 7.43	414.66 ± 17.41
	Particle/mL	1.84x $10^{8}$	9.27x $10^{6}$	2.18x $10^{8}$	3.03x $10^{7}$	1.81x $10^{8}$	5.23x $10^{7}$

#### 3.3.3 nFCM analysis as a promising tool for characterising fluorescent magnetosomes

**FIGURE 6:**
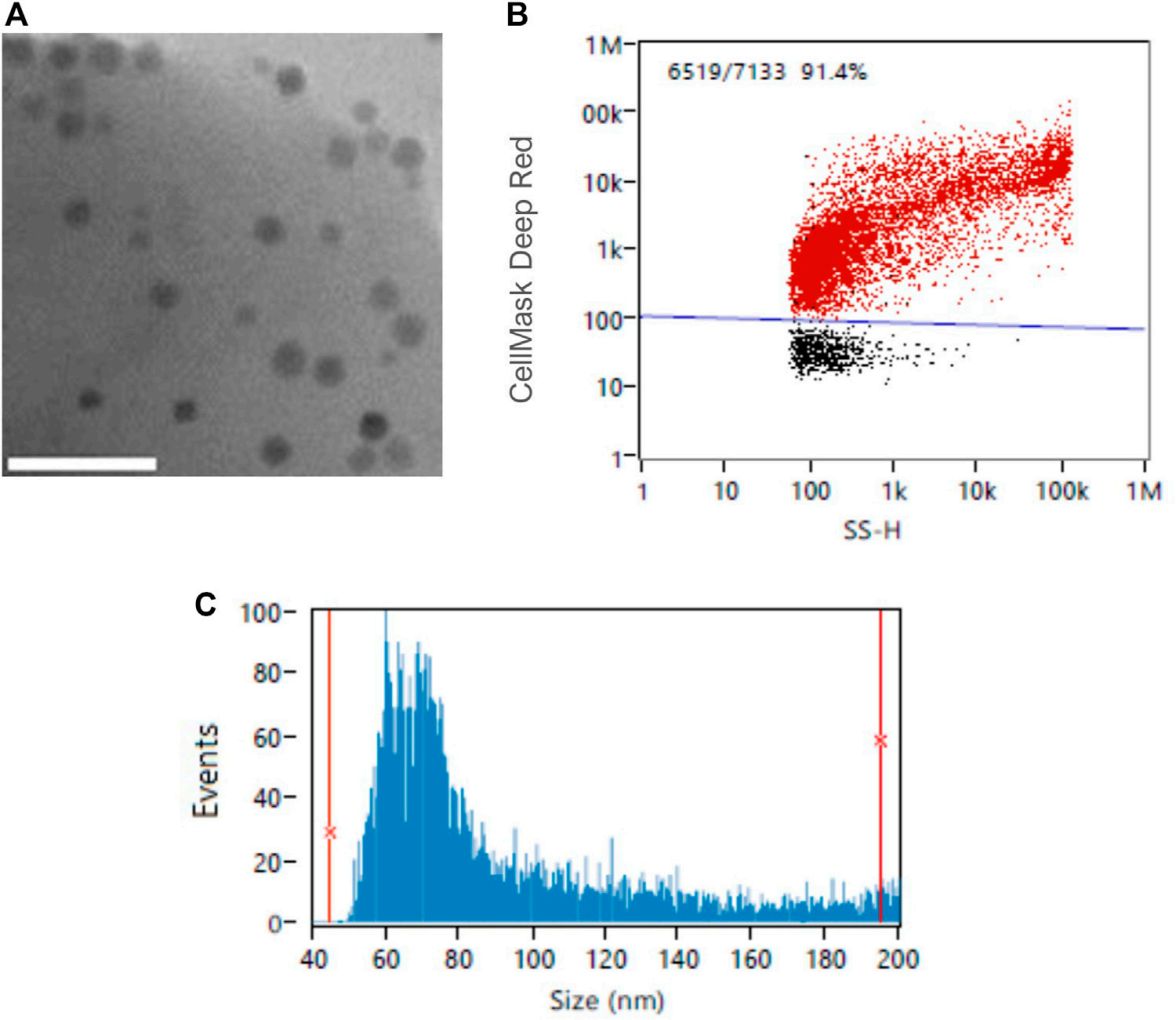
**(A)** Transmission Electron Microscopy (TEM) image of single-chained magnetosomes; Example of **(B)** CellMask™ Deep Red -stained magnetosomes and **(C)** size distribution of single-chained magnetosomes analyzed by NanoFCM Profession V1.8. Scale bar 200 nm.

**FIGURE 7:**
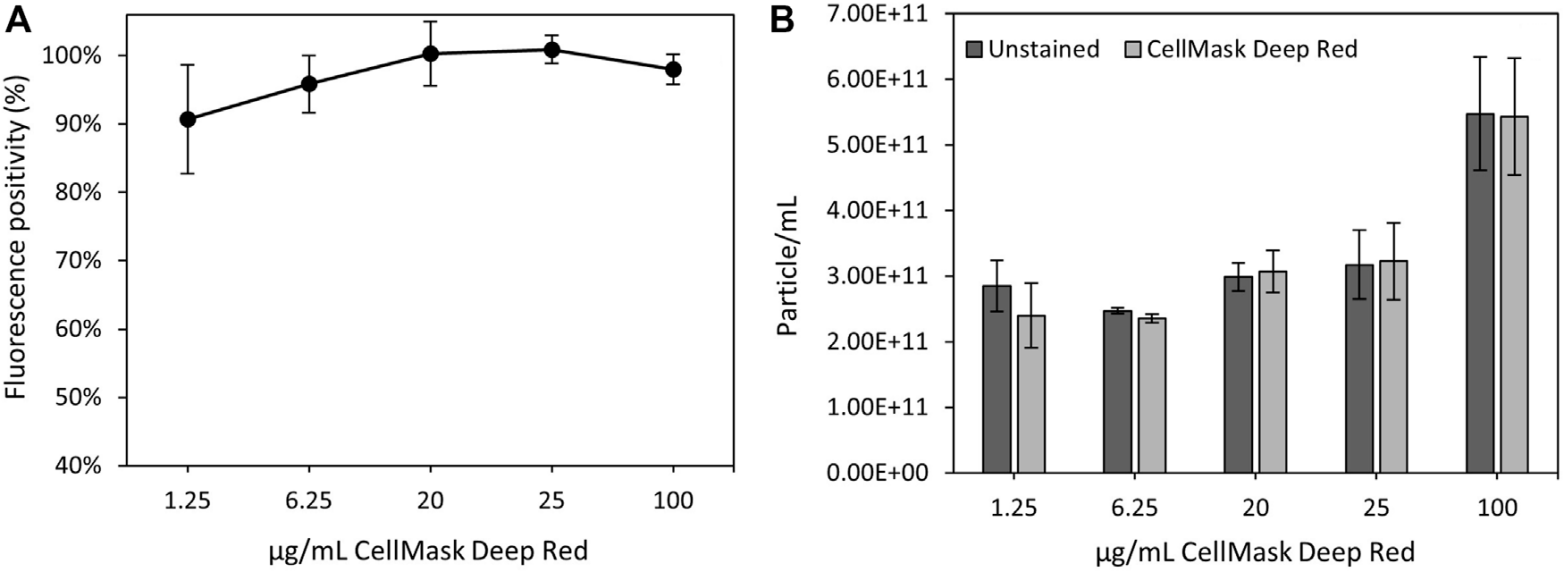
nFCM analysis of magnetosome labelled using CellMask™ Deep Red lipophilic dye. **(A)** Percentage of positive fluorescently labelled magnetosomes using different CellMask™ Deep Red concentrations. **(B)** Particle concentrations of unstained and stained magnetosome solutions.

CellMask™ Deep Reed has been previously used to stain both cells and extracellular vesicle lipidic membranes (Shentu et al., 2017; Aaltonen et al., 2022). In this study, we tested a range of different fluorophore concentrations to determine the optimal magnetosome membrane staining conditions using nFCM technology. It was previously established that nFCM is best suited for single magnetosomes, therefore, the 40–200 nm detection range and single magnetosomes were employed for this analysis (Figure 6). Figure 7A showed the fluorescence positivity of CellMask™ Deep Red stained magnetosomes. Over 90% of the magnetosomes were successfully labelled, and the optimal dye concentration was found to be at 20 μg/mL reaching 100% of stained magnetosomes. For higher concentrations, fluorophore saturation caused a fluorescence positivity decrease (Manoil, 2018). When every molecule is consistently in an excited state, fluorophore saturation can occur, in which an increase in excitation light does not result in a proportional increase in fluorescence emission (Visscher et al., 1994). Figure 7B showed similar particle concentrations between stained and unstained magnetosomes preparations, proving the capacity of CellMask™ Deep Red to specifically stain the magnetosome membrane. The successful detection of labelled magnetosomes shows the promising capacity of this technique to be used as a quality assurance tool for magnetosome preparations. In future studies, being able to directly quantify fluorescently labelled magnetosomes using nFCM will be advantageous for a wide range of different applications (e.g., identification different magnetosome populations, immunofluorescence studies or detection of fluorescent recombinant proteins expressed on the magnetosome surface) (Alphandéry et al., 2018; Mickoleit and Schüler, 2018; Oestreicher et al., 2021).

## 4 Conclusion

Studying the effect of different cell disruption treatments on magnetosome chain length is the key to unlock the potential of magnetosomes to become the next-generation of functional materials. In this study, we systematically compared three optimized disruption techniques for MSR-1 and studied their effects on isolated magnetosome chain length, integrity and aggregation behavior. The results from this study will directly contribute to develop better magnetosome production systems for future applications. Experimental results revealed that EZ treatment caused the highest magnetosome chain breakage whilst HPH best preserved their length. Herein, we have employed nFCM for the characterization of magnetosome for the first time and whereas the tool can be used for quality assurance to determine nanoparticle concentration, we conclude that this novel technology is best suited to characterize single magnetosomes with the particular advantage of being able to reliably determine labelled magnetosomes. Hence nFCM has the potential to become a rapid analytical tool to assess the quality of single magnetosome preparations prior to application studies.

## Data Availability

The original contributions presented in the study are included in the article/Supplementary Material further inquiries can be directed to the corresponding author.

## References

[B1] Aaltonen N. Kyykallio H. Tollis S. Capra J. Hartikainen J. M. Matilainen J. (2022). MCF10CA breast cancer cells utilize hyaluronan-coated EV-rich trails for coordinated migration. Front. Oncol. 12, 869417–869513. 10.3389/fonc.2022.869417 35574334 PMC9091308

[B2] Abdelrazig S. Safo L. Rance G. A. Fay M. W. Theodosiou E. Topham P. D. (2020). Metabolic characterisation of *Magnetospirillum gryphiswaldense* MSR-1 using LC-MS-based metabolite profiling. RSC Adv. 10, 32548–32560. 10.1039/d0ra05326k 35516490 PMC9056635

[B3] Alphandéry E. Abi Haidar D. Seksek O. Guyot F. Chebbi I. (2018). Fluorescent magnetosomes for controlled and repetitive drug release under the application of an alternating magnetic field under conditions of limited temperature increase (<2.5 °C). Nanoscale 10, 10918–10933. 10.1039/C8NR02164C 29850738

[B4] Alphandéry E. Chebbi I. Guyot F. Durand-Dubief M. (2013). Use of bacterial magnetosomes in the magnetic hyperthermia treatment of tumours: A review. Int. J. Hyperth. 29, 801–809. 10.3109/02656736.2013.821527 24024595

[B5] Alphandéry E. Chebbi I. (2012). Preparation of chains of magnetosomes, isolated from *Magnetospirillum magneticum* strain AMB-1 magnetotactic bacteria, yielding efficient treatment of tumors using magnetic hyperthermia. Int. J. Pharm. X. 434, 444–452. 10.1016/j.ijpharm.2012.06.015 22698862

[B6] Alphandéry E. Faure S. Seksek O. Guyot F. Chebbi I. (2011). Chains of magnetosomes extracted from AMB-1 magnetotactic bacteria for application in alternative magnetic field cancer therapy. ACS Nano 5, 6279–6296. 10.1021/nn201290k 21732678

[B7] Alphandéry E. Idbaih A. Adam C. Delattre J. Y. Schmitt C. Guyot F. (2017). Development of non-pyrogenic magnetosome minerals coated with poly-l-lysine leading to full disappearance of intracranial U87-Luc glioblastoma in 100% of treated mice using magnetic hyperthermia. Biomaterials 141, 210–222. 10.1016/j.biomaterials.2017.06.026 28689117

[B8] Amor M. Busigny V. Durand-dubief M. Tharaud M. Ona-nguema G. Gélabert A. (2014). Chemical signature of magnetotactic bacteria. Proc. Natl. Acad. Sci. U. S. A. 4, 1699–1703. 10.1073/pnas.14141121120 PMC4330721 25624469

[B9] Amor M. Ceballos A. Wan J. Simon C. P. Aron A. T. Chang C. J. (2020). Magnetotactic bacteria accumulate a large pool of iron distinct from their magnetite crystals. Appl. Environ. Microbiol. 86, 012788–e1351. 10.1128/aem.01278-20 PMC7642088 32887716

[B10] Bernaerts T. M. M. Gheysen L. Foubert I. Hendrickx M. E. Van Loey A. M. (2019). Evaluating microalgal cell disruption upon ultra high pressure homogenization. Algal Res. 42, 101616. 10.1016/J.ALGAL.2019.101616

[B11] Berny C. Le Fèvre R. Guyot F. Blondeau K. Guizonne C. Rousseau E. (2020). A method for producing highly pure magnetosomes in large quantity for medical applications using *Magnetospirillum gryphiswaldense* MSR-1 magnetotactic bacteria amplified in minimal growth media. Front. Bioeng. Biotechnol. 8, 16. 10.3389/fbioe.2020.00016 32133346 PMC7041420

[B12] Blakemore R. (1975). Magnetotactic bacteria. Sci. (80-. ) 190, 377–379. 10.1126/science.170679 170679

[B13] Bortot B. Mangogna A. Peacock B. Lees R. Valle F. Brucale M. (2022). Platelet activation in ovarian cancer ascites: Assessment of GPIIb/IIIa and PF4 in small extracellular vesicles by nano-flow cytometry analysis | enhanced reader. Cancers (Basel) 14, 4100. 10.3390/cancers14174100 36077635 PMC9454670

[B14] Curcio A. Van De Walle A. Serrano A. Preveral S. Péchoux C. Pignol D. (2020). Transformation cycle of magnetosomes in human stem cells: From degradation to biosynthesis of magnetic nanoparticles anew. ACS Nano 14, 1406–1417. 10.1021/acsnano.9b08061 31880428

[B15] Faivre D. Schüler D. (2008). Magnetotactic bacteria and magnetosomes. Chem. Rev. 108, 4875–4898. 10.1021/cr078258w 18855486

[B16] Falke S. Betzel C. (2019). “Dynamic light scattering (DLS),” in Radiation in bioanalysis (Cham: Springer), 173–193. 10.1007/978-3-030-28247-9_6

[B17] Fernández-Castané A. Li H. Joseph S. Ebeler M. Franzreb M. Bracewell D. G. (2021). Nanoparticle tracking analysis: A robust tool for characterizing magnetosomes preparations. Food Bioprod. Process 127, 426–434. 10.1101/2020.06.23.166587

[B18] Fernández-Castané A. Li H. Thomas O. R. T. Overton T. W. (2018). Development of a simple intensified fermentation strategy for growth of *Magnetospirillum gryphiswaldense* MSR-1: Physiological responses to changing environmental conditions. N. Biotechnol. 46, 22–30. 10.1016/j.nbt.2018.05.1201 29864580 PMC6109776

[B19] Fernández-Castané A. Li H. Thomas O. R. T. Overton T. W. (2017). Flow cytometry as a rapid analytical tool to determine physiological responses to changing O_2_ and iron concentration by *Magnetospirillum gryphiswaldense* strain MSR-1. Sci. Rep. 7, 13118–13211. 10.1038/s41598-017-13414-z 29030621 PMC5640647

[B20] Gojzewski H. Makowski M. Hashim A. Kopcansky P. Tomori Z. Timko M. (2012). Magnetosomes on surface: An imaging study approach. Scanning 34, 159–169. 10.1002/sca.20292 21953296

[B21] Gomes T. A. Zanette C. M. Spier M. R. (2020). An overview of cell disruption methods for intracellular biomolecules recovery. Prep. Biochem. Biotechnol. 50, 635–654. 10.1080/10826068.2020.1728696 32074000

[B22] Grünberg K. Müller E. C. Otto A. Reszka R. Linder D. Kube M. (2004). Biochemical and proteomic analysis of the magnetosome membrane in *Magnetospirillum gryphiswaldense* . Appl. Environ. Microbiol. 70, 1040–1050. 10.1128/AEM.70.2.1040-1050.2004 14766587 PMC348919

[B23] Guo F. Liu Y. Chen Y. Tang T. Jiang W. Li Y. (2011). A novel rapid and continuous procedure for large-scale purification of magnetosomes from *Magnetospirillum gryphiswaldense* . Appl. Microbiol. Biotechnol. 90, 1277–1283. 10.1007/s00253-011-3189-3 21360144

[B24] Hamdous Y. Chebbi I. Mandawala C. Fèvre R. Seksek O. (2017). Biocompatible coated magnetosome minerals with various organization and cellular interaction properties induce cytotoxicity towards RG - 2 and GL - 261 glioma cells in the presence of an alternating magnetic field. J. Nanobiotechnology 1, 74. 10.1186/s12951-017-0293-2 PMC5646109 29041937

[B25] Kleinig A. R. Middelberg A. P. J. (1998). On the mechanism of microbial cell disruption in high-pressure homogenisation. Chem. Eng. Sci. 53, 891–898. 10.1016/S0009-2509(97)00414-4

[B26] Kobayashi A. Kirschvink J. L. Nash C. Z. Kopp R. E. Sauer D. A. Bertani L. E. (2006). Experimental observation of magnetosome chain collapse in magnetotactic bacteria: Sedimentological, paleomagnetic, and evolutionary implications. Earth Planet. Sci. Lett. 245, 538–550. 10.1016/j.epsl.2006.03.041

[B27] Li H. (2018). New bioprocess technologies underpinning future manufacture of magnetosome products. PhD thesis. Birmingham, UK: University of Birmingham.

[B28] Lian H. He S. Chen C. Yan X. (2019). Flow cytometric analysis of nanoscale biological particles and organelles. Annu. Rev. Anal. Chem. (Palo Alto Calif. 12, 389–409. 10.1146/ANNUREV-ANCHEM-061318-115042 30978294

[B29] Makela A. V. Schott M. A. Madsen C. S. Greeson E. M. Contag C. H. (2022). Magnetic particle imaging of magnetotactic bacteria as living contrast agents is improved by altering magnetosome arrangement. Nano Lett. 22, 4630–4639. 10.1021/acs.nanolett.1c05042 35686930

[B30] Mannucci S. Ghin L. Conti G. Tambalo S. Lascialfari A. Orlando T. (2014). Magnetic nanoparticles from *Magnetospirillum gryphiswaldense* increase the efficacy of thermotherapy in a model of Colon Carcinoma. PLoS One 9, e108959. 10.1371/journal.pone.0108959 25289664 PMC4188607

[B31] Manoil D. (2018). Oxidative stress in bacteria measured by flow cytometry. Adv. Biotechnol. Microbiol. 8, 555726. 10.19080/aibm.2018.08.555726

[B32] Mickoleit F. Schüler D. (2018). Generation of multifunctional magnetic nanoparticles with amplified catalytic activities by genetic expression of enzyme arrays on bacterial magnetosomes. Adv. Biosyst. 2, 1700109. 10.1002/ADBI.201700109

[B33] Moisescu C. Ardelean I. I. Benning L. G. (2014). The effect and role of environmental conditions on magnetosome synthesis. Front. Microbiol. 5, 49–12. 10.3389/fmicb.2014.00049 24575087 PMC3920197

[B34] Nakamura N. Hashimoto K. Matsunaga T. (1991). Immunoassay method for the determination of immunoglobulin G using bacterial magnetic particles. Anal. Chem. 63, 268–272. 10.1021/ac00003a015 1824012

[B35] Nanda T. Rathore A. Sharma D. (2020). Biomineralized and chemically synthesized magnetic nanoparticles: A contrast. Front. Mat. Sci. 14, 387–401. 10.1007/s11706-020-0531-7

[B36] Noguchi Y. Fujiwara T. Yoshimatsu K. Fukumori Y. (1999). Iron reductase for magnetite synthesis in the magnetotactic bacterium *Magnetospirillum magnetotacticum* . J. Bacteriol. 181, 2142–2147. 10.1128/jb.181.7.2142-2147.1999 10094692 PMC93627

[B37] Oestreicher Z. Valverde-Tercedor C. Mumper E. Pérez-Guzmán L. Casillas-Ituarte N. N. Jimenez-Lopez C. (2021). Localization of native Mms13 to the magnetosome chain of *Magnetospirillum magneticum* AMB-1 using immunogold electron microscopy, immunofluorescence microscopy and biochemical analysis. Cryst 11, 874. 10.3390/CRYST11080874

[B38] Raguraman V. Jayasri M. A. Suthindhiran K. (2020). Magnetosome mediated oral Insulin delivery and its possible use in diabetes management. J. Mat. Sci. Mat. Med. 31, 75. 10.1007/s10856-020-06417-2 32761252

[B39] Reynoldst J. A. Tanfordt C. (1970). Binding of dodecyl sulfate to proteins at high binding ratios. Possible implications for the state of proteins in biological membranes. Proc. Natl. Acad. Sci. 66, 1002–1007. 10.1073/pnas.66.3.1002 5269225 PMC283150

[B40] Riese C. N. Uebe R. Rosenfeldt S. Schenk A. S. Jérôme V. Freitag R. (2020). An automated oxystat fermentation regime for microoxic cultivation of *Magnetospirillum gryphiswaldense* . Microb. Cell. Fact. 19, 206–215. 10.1186/s12934-020-01469-z 33168043 PMC7654035

[B41] Shentu T. P. Huang T. S. Cernelc-Kohan M. Chan J. Wong S. S. Espinoza C. R. (2017). Thy-1 dependent uptake of mesenchymal stem cell-derived extracellular vesicles blocks myofibroblastic differentiation. Sci. Rep. 71 (7), 18052–18111. 10.1038/s41598-017-18288-9 PMC5741716 29273797

[B42] Souza T. G. F. Ciminelli V. S. T. Mohallem N. D. S. (2016). A comparison of TEM and DLS methods to characterize size distribution of ceramic nanoparticles. J. Phys. Conf. Ser. 733, 012039. 10.1088/1742-6596/733/1/012039

[B43] Sun J. B. Duan J. H. Dai S. L. Ren J. Guo L. Jiang W. (2008). Preparation and anti-tumor efficiency evaluation of doxorubicin-loaded bacterial magnetosomes: Magnetic nanoparticles as drug carriers isolated from *Magnetospirillum gryphiswaldense* . Biotechnol. Bioeng. 101, 1313–1320. 10.1002/bit.22011 18980188

[B44] Suzuki T. Okamura Y. Arakaki A. Takeyama H. Matsunaga T. (2007). Cytoplasmic ATPase involved in ferrous ion uptake from magnetotactic bacterium *Magnetospirillum magneticum* AMB-1. FEBS Lett. 581, 3443–3448. 10.1016/j.febslet.2007.06.047 17618623

[B45] Szatanek R. Baj-Krzyworzeka M. Zimoch J. Lekka M. Siedlar M. Baran J. (2017). The methods of choice for extracellular vesicles (EVs) characterization. Int. J. Mol. Sci. 18, 1153. 10.3390/IJMS18061153 28555055 PMC5485977

[B46] Tang T. Zhang L. Gao R. Dai Y. (2012). Fluorescence imaging and targeted distribution of bacterial magnetic particles in nude mice. Appl. Microbiol. Biotechnol. 94, 495–503. 10.1007/s00253-012-3981-8 22395909

[B47] Tangtua J. (2014). Evaluation and comparison of microbial cells disruption methods for extraction of pyruvate decarboxylase. Available at: https://www.academia.edu/80421983/Evaluation_and_comparison_of_microbial_cells_disruption_methods_for_extraction_of_pyruvate_decarboxylase (Accessed December 16, 2022).

[B48] Ujfalusi-Pozsonyi K. Gabor H. ´Pal G. Zsuzsanna G. T. Zsolt B. Miklos N. (2010). The effects of detergents on the Polymerization Properties of actin. Cytom. part A 77A, 447–456. 10.1002/cyto.a.20855 20151434

[B49] Üstün Aytekin Ö. Arisoy S. Aytekin A. Ö. Yildiz E. (2016). Statistical optimization of cell disruption techniques for releasing intracellular X-prolyl dipeptidyl aminopeptidase from *Lactococcus lactis* spp. Lact. *Ultrason. Sonochem.* 29, 163–171. 10.1016/J.ULTSONCH.2015.09.010 26584994

[B50] Vaghari H. Jafarizadeh-Malmiri H. Mohammadlou M. Berenjian A. Anarjan N. Jafari N. (2015). Application of magnetic nanoparticles in smart enzyme immobilization. Biotechnol. Lett. 38, 223–233. 10.1007/S10529-015-1977-Z 26472272

[B51] Vargas G. Cypriano J. Correa T. Leão P. Bazylinski D. A. Abreu F. (2018). Applications of magnetotactic bacteria, magnetosomes and magnetosome crystals in biotechnology and nanotechnology: Mini-review. Molecules 23, 2438. 10.3390/MOLECULES23102438 30249983 PMC6222368

[B52] Visscher K. Brakenhoff G. J. Visser T. D. (1994). Fluorescence saturation in confocal microscopy. J. Microsc. 175, 162–165. 10.1111/j.1365-2818.1994.tb03479.x

[B53] Wu K. Su D. Liu J. Saha R. Wang J. P. (2019). Magnetic nanoparticles in nanomedicine: A review of recent advances. Nanotechnology 30, 502003. 10.1088/1361-6528/AB4241 31491782

[B54] Xiang L. Wei J. Jianbo S. Guili W. Feng G. Ying L. (2007). Purified and sterilized magnetosomes from *Magnetospirillum gryphiswaldense* MSR-1 were not toxic to mouse fibroblasts *in vitro* . Lett. Appl. Microbiol. 45, 75–81. 10.1111/j.1472-765X.2007.02143.x 17594464

[B55] Yang J. Li S. Huang X. Tang T. Jiang W. Zhang T. (2013). A key time point for cell growth and magnetosome synthesis of *Magnetospirillum gryphiswaldense* based on real-time analysis of physiological factors. Front. Microbiol. 4, 210–217. 10.3389/fmicb.2013.00210 23898327 PMC3721002

[B56] Yoshino T. Hirabe H. Takahashi M. Kuhara M. Takeyama H. Matsunaga T. (2008). Magnetic cell separation using nano-sized bacterial magnetic particles with reconstructed magnetosome membrane. Biotechnol. Bioeng. 101, 470–477. 10.1002/bit.21912 18421798

