# Supplementary material for: Evaluation of cell disruption technologies on magnetosome chain length and aggregation behaviour from Magnetospirillum gryphiswaldense MSR-1
Source: Front Bioeng Biotechnol. 2023 May 4;11:1172457. doi: 10.3389/fbioe.2023.1172457 (PMC10192567; doi:10.3389/fbioe.2023.1172457)
Supplement: Supplementary file 1 [file DataSheet1.DOCX]

Supplementary Material

Evaluation of cell disruption technologies on magnetosome chain length and aggregation behaviour from *Magnetospirillum gryphiswaldense* MSR-1

Marta Masó-Martínez, Benjamin Fryer, Dimitri Aubert, Benjamin Peacock, Rebecca Lees, Graham A Rance, Michael W Fay, Paul D Topham, Alfred Fernández-Castané

*** Correspondence:** Alfred Fernández-Castané: a.fernandez-castane1@aston.ac.uk

# Supplementary Data


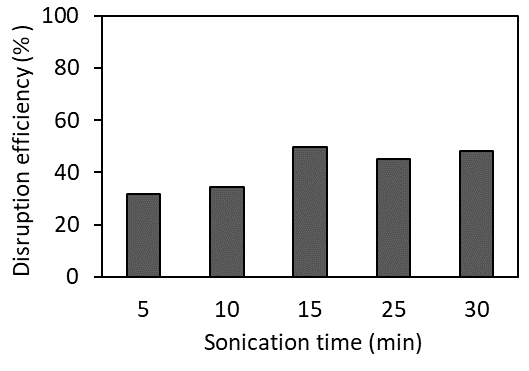


**A**


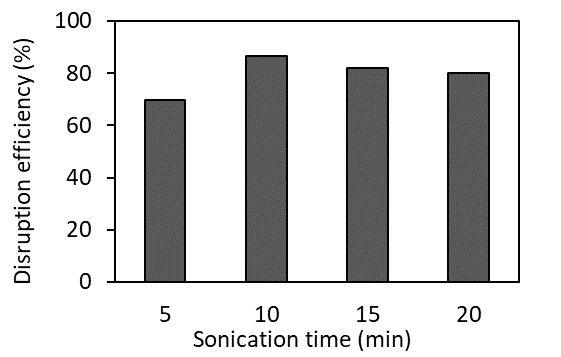


**B**


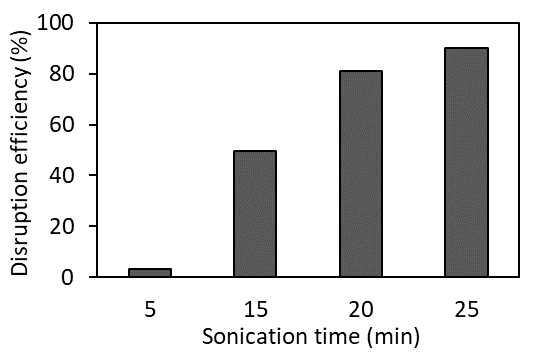


**C**


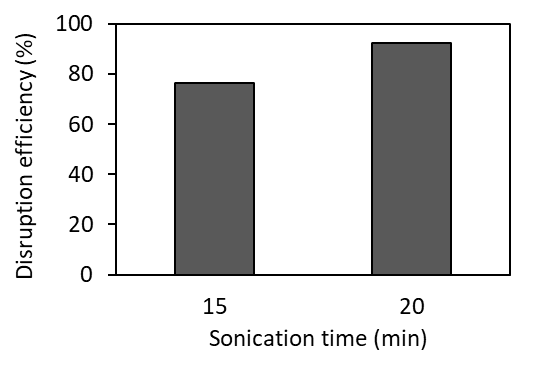


**D**

**Supplementary Figure 1.** Optimization of *M. gryphiswaldense* MSR-1 cell disruption using probe sonication. Different biomass concentrations (10% or 20% (w/v)), sonication pulse settings (1-5 second ON/ 1-5 second OFF), working volumes (1 mL or 20 mL) and sonication times were tested. Disruption efficiency of: (A) 1 mL of 10% WCW biomass with a pulse setting of 5 second ON / 5 second OFF pulse; (B) 1 mL of 10% WCW biomass with a pulse setting of 1 second ON / 1 second OFF pulse; (C) 1 mL of 20% WCW biomass with a pulse setting of 1 second ON / 1 second OFF pulse; (D) 20 mL of 20% WCW biomass with a pulse setting of 1 second ON / 1 second OFF pulse.


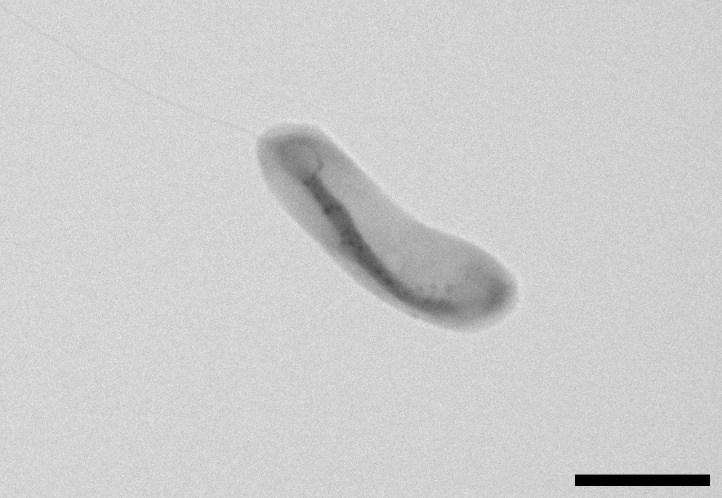

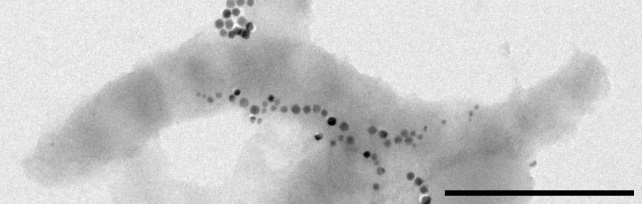

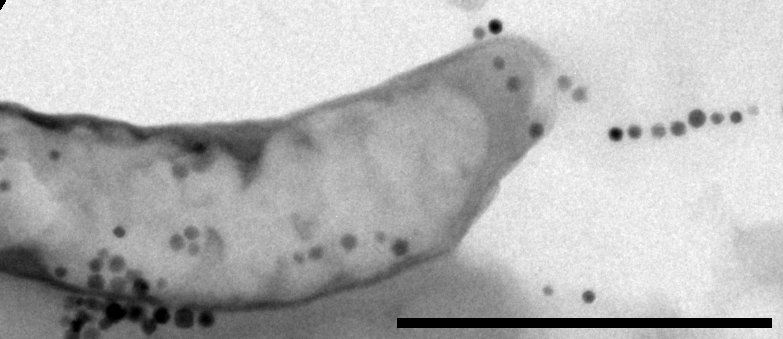


**Supplementary Figure 2.** TEM images of undisrupted *M. gryphiswaldense* MSR-1 cells after enzymatic cell disruption treatment and magnetosome purification. Scale = 1 µm.
